# Developing an Assessment of Contraceptive Preferences in Botswana: Piloting a Novel Approach Using Best-Worst Scaling of Attributes

**DOI:** 10.3389/fgwh.2022.815634

**Published:** 2022-05-18

**Authors:** Alida M. Gertz, Ahmad Syahir Mohd Soffi, Atlang Mompe, Ontiretse Sickboy, Averi N. Gaines, Rebecca Ryan, Aamirah Mussa, Caitlin Bawn, Robert Gallop, Chelsea Morroni, Paul Crits-Christoph

**Affiliations:** ^1^Botswana-University of Pennsylvania Partnership, Gaborone, Botswana; ^2^Botswana Harvard AIDS Institute Partnership, Gaborone, Botswana; ^3^Department of Psychological and Brain Sciences, University of Massachusetts Amherst, Amherst, MA, United States; ^4^Sexual and Reproductive Health Department, Elizabeth Garrett Anderson Institute for Women's Health, University College London, London, United Kingdom; ^5^West Chester University, West Chester, PA, United States; ^6^Medical Research Council (MRC) University of Edinburgh Centre for Reproductive Health, The Queen's Medical Research Institute, Edinburgh, United Kingdom; ^7^Perelman School of Medicine, University of Pennsylvania, Philadelphia, PA, United States

**Keywords:** contraception, family planning, Africa, Botswana, patient preference

## Abstract

**Introduction:**

To develop an attribute-based method for assessing patient contraceptive preferences in Botswana and pilot its use to explore the relationship between patient contraceptive preferences and the contraceptive methods provided or recommended to patients by clinicians.

**Methods:**

A list of contraceptive attributes was developed with input from patients, clinicians, and other stakeholders. We assessed patient preferences for attributes of contraceptives using a discrete choice “best-worst scaling” approach and a multi-attribute decision-making method that linked patient attribute preferences to actual contraceptive method characteristics. Attribute-based patient method preferences and clinician recommendations were compared in 100 women seeking contraceptive services, and 19 clinicians who provided their care. For 41 of the patients, the short-term reliability of their preference scores was also examined.

**Results:**

For 57 patients who wanted more children in the future, the degree of concordance between patients and clinicians was 7% when comparing the top attribute-based contraceptive preference for each woman with the clinician-provided/recommended method. When the top two model-based preferred contraceptive methods were considered, concordance was 28%. For 43 women who did not want more children, concordance was 0% when using the patient's model-based “most-preferred” method, and 14% when considering the top two methods. Assessment of the short-term reliability of preference scores yielded an intraclass correlation coefficient of 0.93.

**Conclusions:**

A best-worst scaling assessment of attributes of contraceptives was designed and piloted in Botswana as a Contraceptive Preference Assessment Tool. The preference assessment was found to have high short-term reliability, which supports its potential use as a measurement tool. There was very low concordance between women's attribute-based contraceptive preferences and their clinician's provision/recommendations of contraceptive methods. Using such a preference assessment tool could encourage greater patient involvement and more tailored discussion in contraceptive consultations.

## Introduction

Preventing unintended pregnancy with effective contraception improves maternal and child health and contributes to the elimination of mother-to-child HIV transmission (1–4). Sub-Saharan Africa has some of the highest levels of unmet need for contraception in the world (5). In Botswana, a country with high maternal mortality (6, 7) and where HIV prevalence is among the highest globally (8), levels of unintended pregnancy remain high, despite gains in contraceptive availability and use (9).

The most commonly used contraceptives in Botswana are shorter-acting methods (condoms, oral contraceptives, and injectables) (10–12). These rely on user adherence, correct use, and regular re-supply; making them less effective under typical use conditions (9, 10). Despite the increasing availability of long-acting reversible methods [LARCS; i.e., implants and intrauterine devices (IUDs)] in Botswana, uptake is limited, and permanent methods are not commonly utilized (10, 11). Recent data show that 43–50% of pregnancies in Botswana are unintended, and 61% of pregnant women desire no further childbearing (9, 12).

To achieve good outcomes with respect to contraceptive method satisfaction and continued use, it is essential that patient preferences be actively incorporated in contraceptive decision-making. Studies report differences between patient and clinician priorities in contraceptive decision-making (13, 14). A systematic review by Wyatt et al. highlighted the discrepancies between attributes included in contraceptive counseling tools and the attributes valued by women (15). An international survey in ten countries found that clinician recommendations to women are influenced by their own personal choice of contraceptive and other factors, such as ease of provision (16).

We aimed to develop a reliable attribute-based method for assessing patient contraceptive preferences in Botswana and to explore the relationship between the contraceptive methods that patients prefer and the methods they are recommended and/or provided. We anticipated a low degree of concordance between patient preferences and clinician-recommendations, due to multiple factors including (1) a tendency toward paternalistic medical practices in contraceptive care, (2) lack of knowledge about different contraceptive methods on the part of both women and providers, and (3) limited time for detailed patient-centered consultations in busy healthcare services. We undertook this preliminary study as a first step toward developing an attribute-based preference assessment tool that could be used to alert clinicians to patient contraceptive preferences in busy and resource-constrained healthcare settings. Such an approach could enable the clinician to prioritize these preferences and attributes during contraceptive counseling. This approach could potentially facilitate patient-clinician conversations about the patient's individual contraceptive preferences and lead to improved patient satisfaction with—and thus more effective use of—contraceptive methods.

## Materials and Methods

In this study, we tested (1) the degree of concordance between patient attribute-based preferences for contraceptive methods and the contraceptive methods their clinicians provided and/or recommended to them (“Concordance Study”), and (2) the short-term reliability of the method preference scores (“Reliability Study”).

### Study Setting

Participant recruitment took place in Gaborone, Botswana at three clinical sites: two Botswana Ministry of Health and Wellness primary care clinics and one NGO-run clinic. Together, these sites are broadly representative of the types of clinics providing contraceptive care to the majority of women in Botswana. During the study period, the three clinical sites had sufficient supply of contraceptives and staff to provide the full range of short- and long-acting reversible contraceptive methods included in the study's contraceptive preference assessment (i.e., male and female condoms, combined oral contraceptive pill, progestogen-only oral contraceptive pill, combined vaginal ring, progestogen-only injectable, intrauterine device, and implant), as well as referral procedures for patients choosing female sterilization. All contraceptive methods were free of charge to receive in these government and NGO clinics at the time of this study.

### Study Participants

#### Concordance Study and Reliability Study

A convenience sample of women who were waiting for family planning services at the clinics were approached for participation. Eligible patients were non-pregnant women aged 18–49, seeking family planning services (seeking initiation, continuation, review, advice, changing or cessation of contraceptive methods or general family planning advice), able to read English/Setswana, and to give informed consent. Women were eligible to participate in the study regardless of their medical eligibility [based on the World Health Organization Medical Eligibility Criteria for contraceptive use (WHO MEC)] for the contraceptive methods included in the preference assessment. After consent, women completed an interviewer-administered demographic, reproductive history, and pregnancy intentions questionnaire and the attribute-based preference assessment before their consultation with a clinician. After each participant's clinical consultation, the attending clinician completed a form that recorded the contraceptive method(s) they had provided and/or recommended to the woman. For this first-stage tool development study, clinicians were not aware of the results of the participant's attribute-based preference assessment. Women provided consent for the study team to keep a secure record of their contact details. A subset of participating women were selected to complete the re-test questionnaire at a later date to assess the short-term reliability of the attribute-based preference assessment. Women participating in the re-test questionnaire were contacted by telephone to arrange a further study visit for ~2 weeks after their initial clinic visit. All participants received 50 Botswana Pula (BWP) compensation, as per Botswana research ethics committee requirements.

### Study Design and Study Components

**Figure 1:**
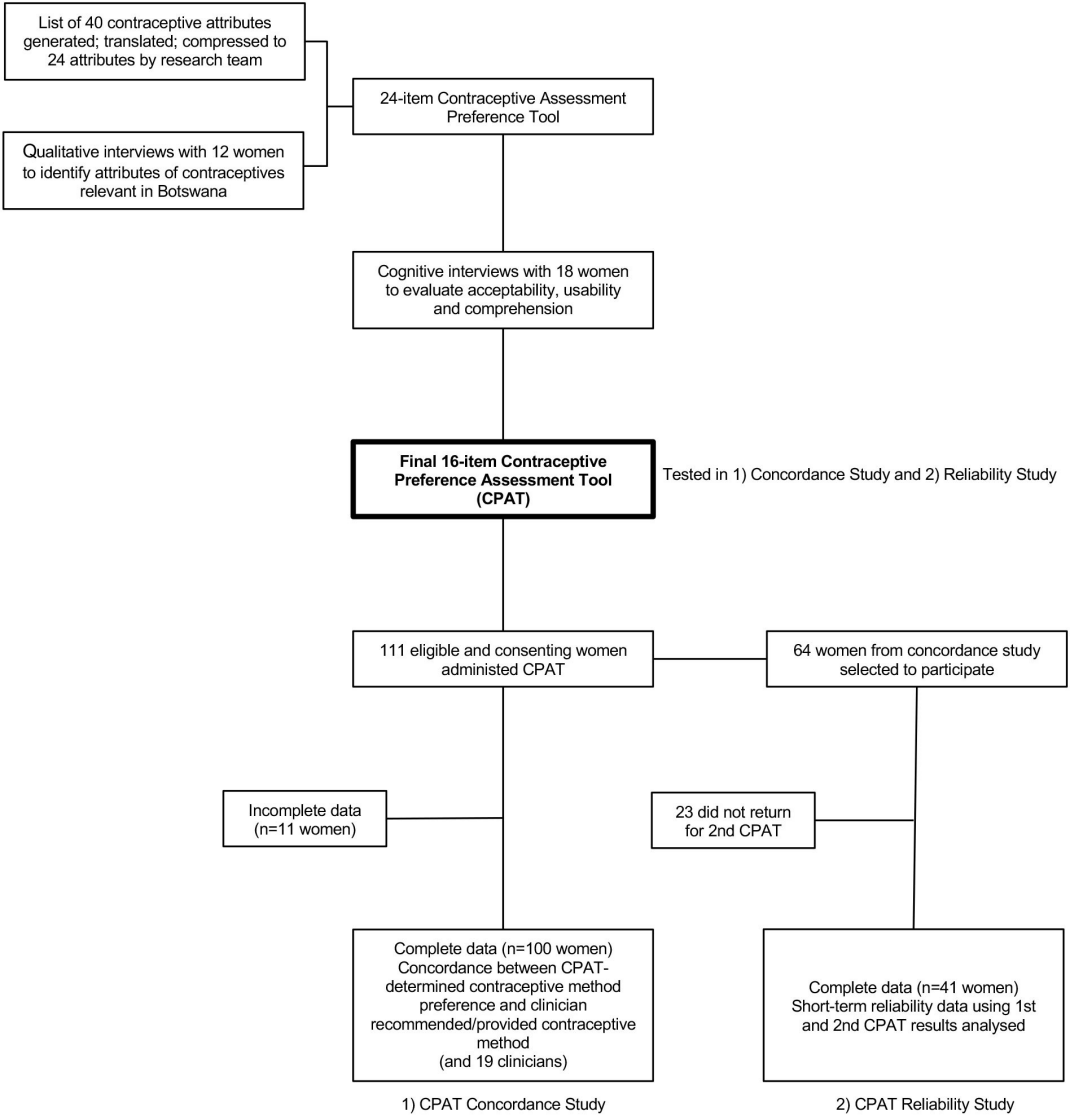
Flow chart of study components for development of a Contraceptive Preference Assessment Tool in Botswana.

See Figure 1 for a flowchart of study components.

#### Development of the Attribute-Based Contraceptive Preference Assessment Tool

An iterative process was used to create a concise yet comprehensive list of contraceptive attributes meaningful to women in Botswana, that would be used for the Preference Assessment. First, a comprehensive list of 40 contraceptive attributes was generated by the research team, in English, using a literature review and qualitative interviews with local stakeholders (comprising twelve women of reproductive age, five clinicians, and three program-officers/family planning nurses from the national family planning program in Botswana). This attributes list was translated into Setswana using forward-back translation and consensus among three qualified translators. Secondly, the attributes were compressed to a more manageable 24-item list. The attributes as well as the wording of the attribute descriptions was informed by three focus group discussions comprised of women of reproductive age and by direct feedback on the list from other local stakeholders to improve the usability of this draft Contraceptive Preference Assessment Tool.

The draft Contraceptive Preference Assessment Tool was then evaluated in 18 women seeking family planning services, for appropriateness, acceptability, usability, and comprehension, including of the best-worst scaling approach, using cognitive interviewing (19–23). This resulted in the further rationalization of the attributes list to 16-items and further refinement of the attribute descriptions (Table 1).

**Table 1:** Final list of 16 contraceptive attributes used in a contraceptive preference assessment tool in Botswana.

1	I want a method that will help me avoid pregnancy at all costs/will be very effective in preventing pregnancy
2	I want to avoid a delay in being able to get pregnant after stopping the method
3	I want a method that will work for a long time
4	I want to use a method privately without other people knowing, for example, my partner or family or friends
5	I want a method that will not require me to remember to use frequently or attend frequent visits to continue using it
6	I want to avoid interruptions during sex to use my method
7	I want to continue having my periods
8	I want less or no periods or bleeding
9	I want to avoid irregular periods/irregular bleeding
10	I want to avoid heavier periods or more painful periods
11	I want to avoid weight gain
12	I want to avoid side effects such as acne, headaches, nausea, breast tenderness, mood changes or lowered sex drive
13	I want to avoid injections or needles
14	I want to avoid a method that requires insertion into my womb
15	I want to avoid a method that requires insertion into my vagina on my own
16	I want a method that also protects me from sexually transmitted infections and HIV

In the three clinics, the final Contraceptive Preference Assessment Tool was presented to eligible and consenting women for the Concordance Study and the Reliability Study via a tablet computer, using the MaxDiff Sawtooth Software (24). This software showed participants four attributes at a time on the tablet computer screen and asked them to designate which of the four attributes were the most and the least important to them (best-worst scaling). Each of the 16 attributes was presented to participants three times in different combinations of four, resulting in a total of 12 questions. The software output was an attribute preference score that quantifies the patient's degree of preference for each contraceptive attribute.

The software output attribute preference score was linked to contraceptive methods with a multiple attribute decision-making (MADM) approach using the Technique for Order Preference by Similarity to Ideal Solution (TOPSIS) method (details of MADM and TOPSIS are provided below). Based on the range of contraceptive methods currently available in Botswana, nine contraceptive methods were included: (1) copper-IUD, (2) male condom, (3) female condom, (4) combined oral contraceptive pill, (5) progestogen-only pill, (6) combined vaginal ring, (7) progestogen-only injectable contraceptive, (8) contraceptive implant, and (9) female sterilization (bilateral tubal ligation).

The TOPSIS model constructs an attribute profile representing an ideal, hypothetical intervention (in this case, a method of contraception) that—if it were to exist—would achieve the patient's desired performance on every attribute.

The attributes of each of the nine contraceptive methods currently available in Botswana were identified by asking three highly experienced contraceptive specialist clinicians to independently score the attributes for each contraceptive method and discuss discrepancies to achieve consensus. For each participant, the TOPSIS model calculated the distance between the ideal, hypothetical contraceptive and each available contraceptive method. The approach also defined an anti-ideal hypothetical option that would achieve the least desirable performance on all attributes. The model computes a preference score (range: 0–100%) for each contraceptive method, which is the distance from the anti-ideal contraceptive divided by the sum of the distance from the anti-ideal contraceptive and the distance from the ideal contraceptive. Using these scores, the available contraceptive methods were rank-ordered for each patient from most to least preferred, according to how well each contraceptive matched the patient's attribute-based preferences. In this study, contraceptive methods ranked as either number one or two were designated as patients' model-predicted “most preferred” contraceptive methods.

### Statistical Analyses

#### Concordance Study Sample Size and Analysis

Based on Cohen's Kappa, a sample of 100 participants is appropriate if 50% agreement is expected, with a 20% error margin (two-tailed alpha 0.05). Thus, we had ample power to detect small effects with 100 patients. However, because we had no previous basis for estimating the degree of concordance (though we expected a low value) and given that the overall concordance rate was analyzed with only descriptive statistics, these sample size calculations were only estimates for planning our study. On an exploratory basis, we tested the statistical significance of differences between clinician-provision/recommendations and patient preferences in the frequency with which individual contraceptive methods occurred among the top options using McNemar's test for paired proportions.

Using the rankings generated by the MADM scores, each woman's model-predicted “preferred” contraceptive method was generated and then compared to the method her clinician provided/recommended. Because female sterilization is a permanent method that would not be recommended to those who may want a child or another child in the future, concordance analyses were conducted separately for those who potentially wanted a child or another child in the future vs. those who did not. Thus, there were nine contraceptive options for those who did not want a future child and eight for those who did. Concordance was calculated as the percentage of patients for whom the model-predicted most “preferred” option was the same as the contraceptive method provided and/or recommended by the clinician.

#### Reliability Study Sample Size and Analysis

Using the formulas of Shoukri et al. (25), we planned a sample size of 40 patients to participate in the Reliability Study to ensure more than 80% power to detect a reliability coefficient of 0.95, with a 95% confidence interval of 0.81–0.99. Of 100 women enrolled in the Concordance Study, 64 were randomly selected to participate in a reliability assessment with the expectation that some attrition would occur between the first and second assessments. For the Reliability Study, MADM scores representing closeness to ideal contraceptive method were calculated for each contraceptive, at the first and second assessments, which took place ~2–4 weeks apart. The relationship between the MADM scores in the two assessments was then evaluated using an intraclass correlation coefficient (ICC) that incorporated the contraceptive options as a repeated-measures factor.

### Ethical Approvals

The Botswana Health Research Development Committee, the University of Botswana Research Ethics Committee, and the Institutional Review Board at the University of Pennsylvania approved the protocol. We obtained written informed consent from all participants.

## Results

### Participant Enrolment and Characteristics

A total of 111 women were enrolled in the Concordance Study and used the Contraceptive Preference Assessment Tool. Preference Assessment Tool data were incomplete for 11 women, leaving a final sample of 100 women for analysis. The completion time of the Contraceptive Preference Assessment Tool by the patient-participants ranged from 15 to 20 min.

All women were attending the clinic for contraceptive initiation, re-supply, method-review, method-switch or contraceptive method concerns. Ninety-eight (98%) women were currently using contraception, most commonly injectables (*n* = 58, 58%) and male condoms (*n* = 24, 24%) (Table 2). Forty-three percent (*n* = 43) of women who enrolled reported they did not want any or more children in the future. Twenty-four women (24%) were living with HIV and all of these women were on antiretroviral medication for HIV treatment. No participant reported World Health Organization Medical Eligibility Criteria (WHO MEC) Category 3 or 4 characteristics or conditions for any of the contraceptive methods included in the study (i.e., no one reported characteristics or conditions that would potentially be contraindications to use of certain contraceptives) (26), meaning all participants were potentially medically eligible for all methods included.

**Table 2:** Characteristics of participants in a study assessing patient contraceptive preferences derived from attribute-based scoring using a Contraceptive Preference Assessment Tool and the degree of concordance between patient preferences for contraceptive methods and clinician recommendations in Botswana.

**Characteristic**	**Do not want (more) children in the future (*n* = 43, 43%)**	**May want (more) children in the future** **(*n* = 57, 57%)**	**Total (*N* = 100)**
**Age**	32.3 (6.5)	26.5 (4.8)	29.0 (6.3)
**Currently in a relationship**	34 (79.1%)	54 (94.7%)	88 (88.0%)
Unmarried, living with partner	11 (25.6%)	17 (29.8%)	28 (28.0%)
Unmarried, not living with partner	13 (30.2%)	34 (59.6%)	47 (47.0%)
Married, living together	9 (20.9%)	2 (3.5%)	11 (11.0%)
Married, not living together	1 (2.3%)	1 (1.8%)	2 (2.0%)
**Employed full or part time**	32 (74.4%)	29 (50.9%)	61 (61.0%)
**Education**			
Primary school	3 (7.0%)	1 (1.8%)	4 (4.0%)
Junior secondary school	15 (34.9%)	23 (40.4%)	38 (38.0%)
Senior secondary school	14 (32.6%)	20 (35.1%)	34 (34.0%)
Tertiary	11 (25.6%)	13 (22.8%)	24 (24.0%)
**Living with HIV**	11 (25.6%)	12 (21.1%)	23 (23.0%)
**Number of living children**	2.53 (1.10)	1.53 (0.86)	1.99 (1.09)
**Current contraceptive method**			
Injectable	27 (62.8%)	31 (54.4%)	58 (58.0%)
Implant	1 (2.3%)	4 (7.0%)	5 (5.0%)
Combined oral contraceptive pill	2 (4.7%)	3 (5.3%)	5 (5.0%)
Progestogen-only pill	2 (4.7%)	0 (0.0%)	2 (2.0%)
Pill (unsure of type)	2 (4.7%)	1 (1.8%)	3 (3.0%)
Copper-IUD	0 (0.0%)	1 (1.8%)	1 (1.0%)
Male condom alone	9 (20.9%)	15 (26.3%)	24 (24.0%)
No method	1 (2.3%)	2 (3.5%)	3 (3.0%)

*Data are represented either as n (%) or mean (standard deviation), as appropriate*.

Nineteen clinicians (17 female; 2 male) were enrolled from three family planning clinics, representing a whole sample of family planning clinicians in those clinics. Most clinicians were midwives (94.7%), and one was a nurse (5.3%). In Botswana, midwives typically provide family planning services. The clinicians reported working in the provision of family planning services for a mean of 18.1 years (standard deviation = 9.9).

### Concordance Study of Patient Preferences and Clinician-Recommendations

Table 3 presents distributions of women's attribute-based model-predicted “preferred” contraceptive methods derived from the Preference Assessment Tool and clinician-provided and/or recommended methods. The distributions for the attribute-based most preferred method (“top 1”) and for the methods that were either the most preferred or the second most preferred (“top 2”) are included in Table 3. For women who wanted the option of having children or more children in the future (*n* = 57), the patient attribute-based scoring from the Preference Assessment Tool suggested that women's model-predicted most “preferred” methods were the LARC methods (94.7%) of copper IUD (84.2%) and implant (10.5%), yet only 29.9% of women were recommended and/or provided with a LARC. Clinicians only provided an IUD to one of these 57 women (1.8%). Clinicians most often provided the injectable (29.8%).

**Table 3:** Clinician-recommended/provided contraceptive methods and patient contraceptive preferences derived from attribute-based scoring using a Contraceptive Preference Assessment Tool among women in Botswana, stratified by those who do or do not want more children in the future.

	**Does not want more children in future (*****n*** **=** **43)**				**May want more children in future (*****n*** **=** **57)**			
	**Clinician- recommended or provided**	**Attribute-score derived patient preference**			**Clinician- recommended or provided**	**Attribute-score derived patient preference**		
		**Top 1**	**Top 2**	*P*-value*		**Top 1**	**Top 2**	*P*-value*
1. Male Condom	0	0	0	1.00	7 (12.3%)	2 (3.5%)	8 (14.0%)	.32
2. Female Condom	0	0	0	1.00	0	2 (3.5%)	8 (14.0%)	.005
3. COC	6 (14.0%)	0	0	0.012	9 (15.8%)	0	0	0.003
4. POP	11 (25.6%)	0	0	0.001	7 (12.3%)	0	0	0.008
5. CVR	0	0	0	1.00	0	0	0	1.00
6. Injectable	16 (37.2%)	0	6 (14.0%)	0.002	17 (29.8%)	1 (1.8%)	15 (26.3%)	0.16
7. IUD	3 (7.0%)	20 (46.5%)	35 (81.4%)	0.001	1 (1.8%)	48 (84.2%)	53 (93.0%)	0.001
8. Implant	7 (16.3%)	0	2 (4.7%)	0.03	16 (28.1%)	6 (10.5%)	36 (63.2%)	0.001
9. Female sterilization (BTL)	0	23 (53.5%)	43 (100%)	0.001	–	–	–	

*Clinician-recommended or provided = number (percentage) of instances in which a clinician recommended each contraceptive method to a patient.*

*Attribute-score derived patient preference = number (percentage) of patients who “preferred” each contraceptive method, with Top 1 indicating that a method was the top ranked, most “preferred” method and Top 2 indicating that the method was identified as either a first or second most “preferred” method (based on best-worst scaling of attribute choices). Female sterilization (BTL) option was not considered as an option for those who potentially wanted more children.*

* *P-value for contrasts between clinician-recommended and patient preference is a comparison based on clinician vs. Top 2 and assessed through McNemar's test for paired proportions.*

*COC, combined oral contraceptive; POP, progestogen-only pill; CVR, combined vaginal ring; BTL, bilateral tubal ligation*.

Among women who stated they did not want more children (*n* = 43), attribute-based scoring derived from the Preference Assessment Tool suggested the model-predicted most “preferred” method was female sterilization (53.5%), followed by the copper IUD (46.5%). Clinicians recommended these methods to patients 0 and 7% of the time, respectively.

Whilst the model-predicted most preferred method for all women in this group was a LARC or permanent method, only 23.3% of women were provided with or recommended one; the rest received or were recommended shorter-acting methods. For these 43 women, the most frequent clinician-recommended options were injectables (37.2%) and the progestogen-only pill (25.6%).

The frequency of contraceptive methods that occurred within the top two attribute-based methods for patients was significantly different to the frequency based on clinician recommendations (Table 3).

For the 57 patients who reported they potentially wanted more children in the future, the overall degree of concordance between patient preferences for contraceptive methods and the methods their clinicians provided/recommended was 7% when comparing the top choice, and 28.1% when the top two preferences were considered for each woman.

Similarly, for the 43 women who did not want more children, the degree of concordance was 0% when using the patient's model-predicted most preferred method, and 14% when considering the top two model-predicted methods for each patient.

### Short-Term Reliability Study

Forty-one of the 64 participants selected for the Reliability Study completed the second preference assessment. These 41 participants were similar to the 64 selected and to the total cohort (data not shown).

The median time between the first and second preference assessments was 16 days (range: 8–34 days). The comparison of MADM scores between assessment one and assessment two (*n* = 41) showed a high degree of short-term reliability of the Contraceptive Preference Assessment Tool derived preference scores with an ICC of 0.93 (95% CI: 0.91–0.94).

## Discussion

There are three main findings from our study. The first is that a best-worst scaling assessment of contraceptive attributes was designed and successfully piloted in Botswana. The preference assessment was designed to be understandable and efficient for use among women seeking family planning care in Botswana, a low- and middle-income country (LMIC) setting with a resource-constrained health service. Our second finding was that the preference assessment had high short-term reliability, which supports its potential use as a measurement tool within research and an clinical settings. The third finding was that, as we hypothesized, there was very low concordance between women's contraceptive preferences and their clinician's provision of and recommendations for contraceptive methods.

The limited concordance between women's modeled attributes-based contraception preferences and clinician provision and/or recommendations highlights the need for better assessment and consideration of women's preferences during contraceptive consultations and greater shared decision-making between women and clinicians in Botswana, particularly in relation to longer-acting and permanent methods (which commonly matched women's model-predicted preferred attributes). Previous studies have shown that clinicians play a key role in influencing women's choice of contraception (27, 28), and while women ultimately want control over contraceptive decision-making based on their own values and preferences, there is also a desire for their clinician to be involved in the decision-making process (29).

Some existing approaches such as the My Contraceptive Tool (17) and My Birth Control (18) also assess patient preferences for contraceptive attributes as part of a decision-making tool, but these have not been developed or assessed in an African setting. Our approach differs from these in that our attribute-based method was designed as a brief assessment tool that could potentially be administered quickly in resource- and time-limited settings. The use of best-worst scaling to measure preferences between different contraceptive attributes could potentially be more efficient than relying solely on an importance rating of each attribute, better differentiate patients' most preferred option(s), and enable the clinician to better prioritize patient preferences during contraceptive counseling.

Although the current study demonstrated that an attribute-based assessment of patient contraceptive preferences could be developed, the methods used did not allow for real-time clinical decision-making. We plan to create a software app to capture the preference ranking in real-time for testing in prospective studies and eventual clinical use in contraceptive consultations.

A previous cluster randomized trial evaluating the impact of a contraceptive preference decision-aid in the U.S. did not show any significant effects on contraceptive continuation or satisfaction with the contraceptive method (18). The intervention did, however, enhance the experience of contraceptive counseling and informed decision-making, and led to greater levels of knowledge related to contraception (18). The lack of effect on contraceptive continuation in the U.S. may not hold in countries like Botswana, where knowledge of contraceptives is more limited and stark discrepancies between healthcare recommendations and patient preferences are prevalent. Randomized trials of such decision aids in African and LMIC settings are needed to evaluate this.

Our study had limitations. As a single-country study, results may not be applicable to other settings with different cultural norms and patterns of contraceptive use. Adolescents under the age of 18 years were not included in our study, as parental consent is required for this age group. Adolescents are an important group in need of contraceptive services, and it would be important to ensure the Preference Assessment Tool also works for young people in further work. Women and providers may also have reasons outside of pregnancy prevention for choosing or not choosing specific contraceptive methods, and this tool was not designed to capture that (30–32). Our preference assessment took up to 20 min to complete and required literacy, which may limit in-field use as a decision-making tool, so further research on usability and necessary adaptations for wider use are needed. Further, it did not include an information-giving component on contraceptive methods, which has been included in some other tools and may enhance utility as it is further developed.

In conclusion, the current study provides preliminary information on the development of a novel assessment approach using best-worst scaling of attributes to measure patient preferences for contraceptive methods. In addition, we have demonstrated a general lack of concordance between the preferences of women in Botswana seeking contraceptive care and the methods clinicians recommend and/or provide to them. Further and more in-depth research is needed to examine whether patient preference assessment tools better incorporate patient preferences into contraceptive care, and in turn, improve long-term patient satisfaction, adherence to family planning methods, and reductions in unintended pregnancies in Botswana and other similar settings.

## Data Availability Statement

The raw data supporting the conclusions of this article will be made available by the authors, without undue reservation.

## Ethics Statement

The studies involving human participants were reviewed and approved by Botswana Health Research Development Committee, University of Botswana Ethics Committee and Institutional Review Board at the University of Pennsylvania. The patients/participants provided their written informed consent to participate in this study.

## Author Contributions

CM and PC-C were involved with conception and design. CM, AS, AMG, AMo, AMu, OS, and ANG were involved with the implementation of the study. RG and PC-C performed the statistical analysis. CM, PC-C, AMu, and RR drafted the manuscript. All authors have read and approved the manuscript.

## Funding

Research reported in this publication was supported by the Bill and Melinda Gates Foundation (grant# OPP1161549) and the National Institute of Mental Health (grant R21-MH108996). AG's time was supported by the Fogarty International Center and National Institute of Mental Health, of the National Institutes of Health under Award Number D43 TW010543, and the Afya Bora Consortium Fellowship program.

## Author Disclaimer

The content is solely the responsibility of the authors and does not necessarily represent the official views of the Bill and Melinda Gates Foundation or the National Institute of Mental Health.

## Conflict of Interest

The authors declare that the research was conducted in the absence of any commercial or financial relationships that could be construed as a potential conflict of interest.

## Publisher's Note

All claims expressed in this article are solely those of the authors and do not necessarily represent those of their affiliated organizations, or those of the publisher, the editors and the reviewers. Any product that may be evaluated in this article, or claim that may be made by its manufacturer, is not guaranteed or endorsed by the publisher.

## References

[1] UNFPA. Preventing HIV and Unintended Pregnancies: Strategic Framework 2011–2015. (2016). Available online at: http://www.unfpa.org/sites/default/files/pub-pdf/V2_web_P1P2_framework 22.8.12.pdf (accessed March 26, 2020).

[2] Hubacher D Mavranezouli I McGinn E. Unintended pregnancy in sub-Saharan Africa: magnitude of the problem and potential role of contraceptive implants to alleviate it. Contraception. (2008) 78:73–8. 10.1016/j.contraception.2008.03.002 18555821

[3] Reynolds HW Janowitz B Homan R Johson L. The value of contraception to prevent perinatal HIV transmission. Sex Transm Dis. (2006) 33:350–6. 10.1097/01.olq.0000194602.01058.e1 16505747

[4] Reynolds HW Janowitz B Wilcher R Cates W. Contraception to prevent HIV-positive births: current contribution and potential cost savings in PEPFAR countries. Sex Transm Infect. (2008) 84 (Suppl. 2):ii49–53. 10.1136/sti.2008.030049 18799493

[5] Singh S DJ. Adding It Up: Costs Benefits of Contraceptive Services—Estimates for 2012. New York Guttmacher Inst. United Nations Popul. Fund. (2012). Available online at: https://www.guttmacher.org/sites/default/files/report_pdf/aiu-2012-estimates_0.pdf (accessed March 26, 2020).

[6] Statistics Botswana. Botswana - Maternal Mortality Ratio 2017. (2017). Available online at: http://www.statsbots.org.bw/sites/default/files/publications/Botswana Martenal Mortality Ratio 2017.pdf (accessed March 28, 2020).

[7] Sinvula M Insua M. Botswana Maternal Mortality Reduction Initiative. (2015). Available online at: https://www.usaidassist.org/sites/default/files/botswana_mmri_final_report_dec2015.pdf (accessed March 28, 2020).

[8] UNAIDS. AIDSinfo: HIV Prevalence. (2018). Available online at: http://aidsinfo.unaids.org (accessed March 28, 2020).

[9] Mayondi GK Wirth K Morroni C Sikhulile M Ajibola G Diseko M. Unintended pregnancy, contraceptive use, and childbearing desires among HIV-infected and HIV-uninfected women in Botswana: a cross-sectional study. BMC Public Health. (2016) 16:44. 10.1186/s12889-015-2498-3 26774918 PMC4715872

[10] World Bank. Reproductive Health at a Glance: Botswana. (2011). Available online at: http://documents.worldbank.org/curated/en/618401468201249044/pdf/629170BRIEF0Bo0BOX0361514B00PUBLIC0.pdf (accessed March 26, 2020).

[11] Central Statistics Office. Botswana Family Health Survey IV Report (2007).

[12] Doherty K Arena K Wynn A Offorjebe OA Moshashane M Sickboy O. Unintended pregnancy in Gaborone, Botswana: a cross-sectional study. Afr J Reprod Health. (2018) 22:76–82. 10.29063/ajrh2018/v22i2.8 30052336

[13] Weisberg E Bateson D Knox S Haas M Viney R Street D. Do women and providers value the same features of contraceptive products? Results of a best-worst stated preference experiment. Eur J Contracept Reprod Heal Care. (2013) 18:181–90. 10.3109/13625187.2013.777830 23557397

[14] Donnelly KZ Foster TC Thompson R. What matters most? The content and concordance of patients' and providers' information priorities for contraceptive decision making. Contraception. (2014) 90:280–7. 10.1016/j.contraception.2014.04.012 24863169

[15] Wyatt KD Anderson RT Creedon D Montori VM Bachman J Erwin P. Women's values in contraceptive choice: a systematic review of relevant attributes included in decision aids. BMC Womens Health. (2014) 14:28. 10.1186/1472-6874-14-28 24524562 PMC3932035

[16] Gemzell-Danielsson K Cho S Inki P Mansour D Reid R Bahamondes L. Use of contraceptive methods and contraceptive recommendations among health care providers actively involved in contraceptive counseling — results of an international survey in 10 countries. Contraception. (2012) 86:631–8. 10.1016/j.contraception.2012.06.002 22770797

[17] French RS Cowan FM Wellings K Dowie J. The development of a multi-criteria decision analysis aid to help with contraceptive choices: my contraception tool. J Fam Plan Reprod Heal care. (2014) 40:96–101. 10.1136/jfprhc-2013-100699 24265469

[18] Dehlendorf C Fitzpatrick J Fox E Holt K Vittinghoff E Reed R. Cluster randomized trial of a patient-centered contraceptive decision support tool, my birth control. Am J Obstet Gynecol. (2019) 220:565.e1-565.e12. 30763545 10.1016/j.ajog.2019.02.015

[19] Finn A Louviere JJ. Determining the appropriate response to evidence of public concern: the case of food safety. J Public Policy Mark. (1992) 11:12–25. 10.1016/j.ajog.2019.02.015 30763545

[20] Flynn TN Louviere JJ Peters TJ Coast J. Estimating preferences for a dermatology consultation using best-worst scaling: comparison of various methods of analysis. BMC Med Res Methodol. (2008) 8:76. 10.1186/1471-2288-8-76 19017376 PMC2600822

[21] Louviere JJ Flynn TN Marley AAJ. Best-Worst Scaling. Cambridge: Cambridge University Press (2015).

[22] Marley AAJ Flynn TN Louviere JJ. Probabilistic models of set-dependent and attribute-level best–worst choice. J Math Psychol. (2008) 52:281–96. 10.1016/j.jmp.2008.02.002

[23] Marley AAJ Louviere JJ. Some probabilistic models of best, worst, and best–worst choices. J Math Psychol. (2005) 49:464–80. 10.1016/j.jmp.2005.05.003

[24] Sawtooth Software. MaxDiff. Available online at: https://www.sawtoothsoftware.com/products/maxdiff-software (accessed April 14, 2020).

[25] Shoukri MM Asyali MH Donner A. Sample size requirements for the design of reliability study: review and new results. Stat Methods Med Res. (2004) 13:251–71. 10.1191/0962280204sm365ra33894684

[26] World Health Organization. Medical Eligibility Criteria for Contraceptive Use. 5th ed. (2015). Available online at: https://apps.who.int/iris/bitstream/handle/10665/181468/9789241549158_eng.pdf?sequence=9 (accessed April 20, 2020).

[27] Bitzer J Gemzell-Danielsson K Roumen F Marintcheva-Petrova M van Bakel B Oddens BJ. The CHOICE study: effect of counselling on the selection of combined hormonal contraceptive methods in 11 countries. Eur J Contracept Reprod Heal Care. (2012) 17:65–78. 10.3109/13625187.2011.637586 22239264

[28] Johnson S Pion C Jennings V. Current methods and attitudes of women towards contraception in Europe and America. Reprod Health. (2013) 10:7. 10.1186/1742-4755-10-7 23384291 PMC3599328

[29] Dehlendorf C Levy K Kelley A Grumbach K Steinauer J. Women's preferences for contraceptive counseling and decision making. Contraception. (2013) 88:250–6. 10.1016/j.contraception.2012.10.012 23177265 PMC4026257

[30] Chiofalo B Laganà AS Imbesi G Vitale SG Catena U Campolo F. Is oral contraceptive-induced headache dependent on patent foramen ovale? Clinical dynamics, evidence-based hypothesis and possible patient-oriented management. Med Hypotheses. (2016) 94:86–8. 10.1016/j.mehy.2016.07.003 27515209

[31] Laganà AS Vitale SG Granese R Palmara V Ban FrangeŽ H Vrtačnik-Bokal E. Clinical dynamics of Dienogest for the treatment of endometriosis: from bench to bedside. Expert Opin Drug Metab Toxicol. (2017) 13:593–6. 10.1080/17425255.2017.1297421 28537213

[32] Sansone A De Rosa N Giampaolino P Guida M Laganà AS Di Carlo C. Effects of etonogestrel implant on quality of life, sexual function, and pelvic pain in women suffering from endometriosis: results from a multicenter, prospective, observational study. Arch Gynecol Obstet. (2018) 298:731–6. 10.1007/s00404-018-4851-0 30074068

